# A new approach to biomining: Bioengineering surfaces for metal recovery from aqueous solutions

**DOI:** 10.1038/s41598-019-52778-2

**Published:** 2019-11-11

**Authors:** Jesica Urbina, Advait Patil, Kosuke Fujishima, Ivan G. Paulino-Lima, Chad Saltikov, Lynn J. Rothschild

**Affiliations:** 1 University of California Santa Cruz, Department of Microbiology and Environmental Toxicology, Santa Cruz, CA 95064 USA; 2 Universities Space Research Association, Mountain View, CA 94043 USA; 3 Tokyo Institute of Technology, Earth-Life Science Institute (ELSI), Tokyo, Japan; 4 Blue Marble Space Institute of Science, Seattle, Washington, 98154 USA; 5 NASA Ames Research Center, Space Biosciences Division, Moffett Field, CA 94035 USA

**Keywords:** Biomaterials - proteins, Environmental biotechnology

## Abstract

Electronics waste production has been fueled by economic growth and the demand for faster, more efficient consumer electronics. The glass and metals in end-of-life electronics components can be reused or recycled; however, conventional extraction methods rely on energy-intensive processes that are inefficient when applied to recycling e-waste that contains mixed materials and small amounts of metals. To make e-waste recycling economically viable and competitive with obtaining raw materials, recovery methods that lower the cost of metal reclamation and minimize environmental impact need to be developed. Microbial surface adsorption can aid in metal recovery with lower costs and energy requirements than traditional metal-extraction approaches. We introduce a novel method for metal recovery by utilizing metal-binding peptides to functionalize fungal mycelia and enhance metal recovery from aqueous solutions such as those found in bioremediation or biomining processes. Using copper-binding as a proof-of-concept, we compared binding parameters between natural motifs and those derived *in silico*, and found comparable binding affinity and specificity for Cu. We then combined metal-binding peptides with chitin-binding domains to functionalize a mycelium-based filter to enhance metal recovery from a Cu-rich solution. This finding suggests that engineered peptides could be used to functionalize biological surfaces to recover metals of economic interest and allow for metal recovery from metal-rich effluent with a low environmental footprint, at ambient temperatures, and under circumneutral pH.

## Introduction

End-of-life electronics waste (e-waste) production has been fueled by economic growth and the demand for faster, more efficient consumer electronics. The glass and metals in e-waste can be reused or recycled however, developed countries tend to not recycle due to high labor costs and strict environmental regulation^1^. Instead, e-waste accumulates in landfills or is exported to developing countries where it is recycled using primitive techniques such as open-air incineration or strong acid treatments for metal recovery, without regard for worker safety or environmental impact^2,3^. Additionally, many elemental components in e-waste such as the rare-earth elements (REEs) and transition metals like titanium (Ti) are emerging as new contaminants that have never before existed in concentrated quantities sufficient to produce toxicity to organisms^4–6^. These activities have led to severe heavy metal pollution in communities that handle e-waste as they are experiencing adverse health effects and toxicity to aquatic and terrestrial ecosystems^7,8^. Development of economically viable alternatives for elemental recovery is essential for sustaining a balance between technological development and environmental responsibility.

Conventional extraction methods rely on energy-intensive processes and are inefficient when applied to recycling e-waste that contains mixed materials and small amounts of metals. Applying a biological approach to resource extraction from e-waste, which we term *urban biomining*, allows for metal extraction at ambient temperatures with lower environmental impacts and energy requirements than current approaches. Microbial surface adsorption can aid in metal recovery from aqueous solutions containing metals from e-waste however, there is limited specificity because the surface functional groups will bind many cations with high affinity^9,10^. A handful of studies have focused on the addition of metal-binding peptide tags onto bacterial surface proteins and they have shown to sequester more metal than controls however, the tags offer limited specificity as to the metals that are adsorbed^11–15^.

In principle, a biological approach to metal adsorption using peptides should be exquisitely specific because all cellular fluids contain a mixture of metal ions at different concentrations, yet metal cofactors are not easily replaced from their cognate metalloproteins by competing ions in the intracellular milieu. Metal coordination number (the ability to bind to a given number of ligands) and molecular geometry are shared properties between a ligand and its cognate metal and are proposed to be a key determinant of specificity^16^. A metalloprotein will bind a metal cofactor with amino acids in the primary coordination sphere that refers to the molecules that are attached directly to the metal, and that is optimal for the molecular geometry and coordination number of the cognate metal. The effects of the second and third coordination spheres consist of molecules that interact via hydrogen and Van der Waals interactions with the primary coordination sphere and are those presumed to determine specificity for a metal co-factor. Computer-generated design that considers only the primary coordination sphere of a metal and the approximate steric compatibility of a scaffold protein can be used to introduce a selective binding site into a protein^17,18^. Yet, it remains challenging to model metal specificity into *de novo*-designed proteins due to substantial computational requirements^19,20^. An alternative to computational design is a biopanning technique to identify metal-binding peptides that are selective for a target metal and have also shown promise in identifying natural proteins that adsorb target metals^21,22,25^.

For this work, we hypothesized that a metal-binding motif from the primary coordination sphere of a metalloprotein (natural or designed *in silico*) was sufficient to bind a metal (Cu) with high affinity and fidelity without regard for the secondary or tertiary coordination spheres. Our objective was to develop an approach for biorecovery of metals from aqueous solutions using metal-binding peptides to adsorb various metals of economic and geopolitical interest from a metal-rich leachate solution. First, we compare naturally-occurring metal binding motifs to those designed *in silico*, to elucidate factors in binding and specificity that can be applied to other metals. Since the role and chemistry of copper (Cu) in biology is relatively well-characterized, we use this as a proxy for metals with unknown biological roles such as REEs or platinum group metals. Second, we show a pathway to implementation by demonstrating through pilot experiments that the application of our metal-binding peptides containing a chitin-binding domain can functionalize the otherwise hydrophobic surface of fungal mycelia to aid in metal recovery. Our data indicate that natural and designer motifs are selective and effective metal chelators, and that when these are used to functionalize a mycelial surface, they increase the metal-binding capacity of an inexpensive and non-toxic substrate that can be used in metal recovery applications. In order for e-waste recycling to be economically viable and competitive with raw materials, recovery methods that lower the cost of metal reclamation and minimize environmental impact need to be developed.

## Results

### Intrinsic binding parameters for Cu

To compare binding affinities from natural motifs versus those derived *in silico*, we characterized select peptides for their binding parameters through isothermal titration calorimetry (ITC). The natural peptides were chosen because they have been previously characterized and have amino acid residues that are highly represented in Cu-binding. The putative Cu-binding domain CXXC, is found in metalloproteins with diverse functions such as the metal-binding domain of *E. coli* GTPase (HypB1,2)^23,24^, *Arabidopsis sp*. Zn- and Cu- binding peptides (CZB-7)^25^, and in a consensus motif represented in different types of Cu binding (Cu-02)^26^. The rationally-designed peptide motifs we used for comparison are predicted, *in silico*, to bind Cu (HHTC, CHSK) or Zn (KDKD, KDTK)^27^. The peptides were derived by applying quantum mechanical methods that consider complexation energies of the metal with the amino acid side chains of a primary coordination sphere in a metalloprotein, and the molecular geometry (Ni - octahedral, Zn - tetrahedral, Cu - square planar) of the cognate metal^27^. These are referred to according to their metal binding residues. We used a positive control, HHTC, that was previously characterized for Cu binding by using matrix-assisted desorption/ionization (MALDI) and isothermal titration calorimetry (ITC), and CHSK as a negative control determined through MALDI to not bind Cu in the gas phase despite *in silico* predictions. The complete list of assessed peptides and their respective amino acid sequences is in Table 1.

**Table 1:** Metal-binding motifs assessed for Cu-binding parameters. Intrinsic association constants (K_a_) for peptides titrated with Cu, Zn, Ni, and apparent binding constants when titrated with Cu after Zn or Ni was in solution.

Type	Name	Amino acid sequence	Cognate metal	Source	Cu [M^−1^]	Zn [M^−1^]	Ni [M^−1^]	Zn → Cu [M^−1^]	Ni → Cu [M^−1^]
Natural motifs	HypB1	CTTCGCG	Unknown	Douglas *et al*., 2012	(2.37 ± 0.71) × 10^6^	0	0	(1.29 ± 0.26) × 10^7^	n/a
	HypB2	MCTTCGCGEG	Unknown	Chang *et al*., 2008	(1.30 ± 0.07) × 10^6^	0	0	(1.98 ± 0.99) × 10^6^	(3.51 ± 0.18) × 10^6^
	CZB-7	GFHGRADALLHKI	Cu/Zn	Yeh *et al*., 2010	(7.78 ± 1.25) × 10^3^	0	0	0	0
Consensus	Cu-02	HCWCHM	Cu	Bertini *et al*., 2010	(9.89 ± 2.18) × 10^5^	0	0	(4.97 ± 0.7) × 10^4^	0
Rational design	HHTC	HNLGMNHDLQGERPYVTEGC	Cu	Kozisek *et al*., 2008	(1.74 ± 0.49) × 10^6^	0	0	(8.64 ± 3.11) × 10^5^	(5.02 ± 1.10) × 10^5^
	CHSK	CPSEDHVSQDK	Cu		(1.28 ± 0.33) × 10^6^	0	0	(2.35 ± 0.81) × 10^5^	0
	KDTK	KTEYVDERSKSLTVDLTK	Zn		(1.05 ± 0.91) × 10^4^	(2.44 ± 3.53) × 10^3^	0	(6.51 ± 0.75) × 10^3^	n/a
	KDKD	KFFKDFRHKPATELTHED	Zn		(1.27 ± 0.11) × 10^4^	0	0	(3.08 ± 2.33) × 10^6^	(1.71 ± 1.08) × 10^6^

**Figure 1:**
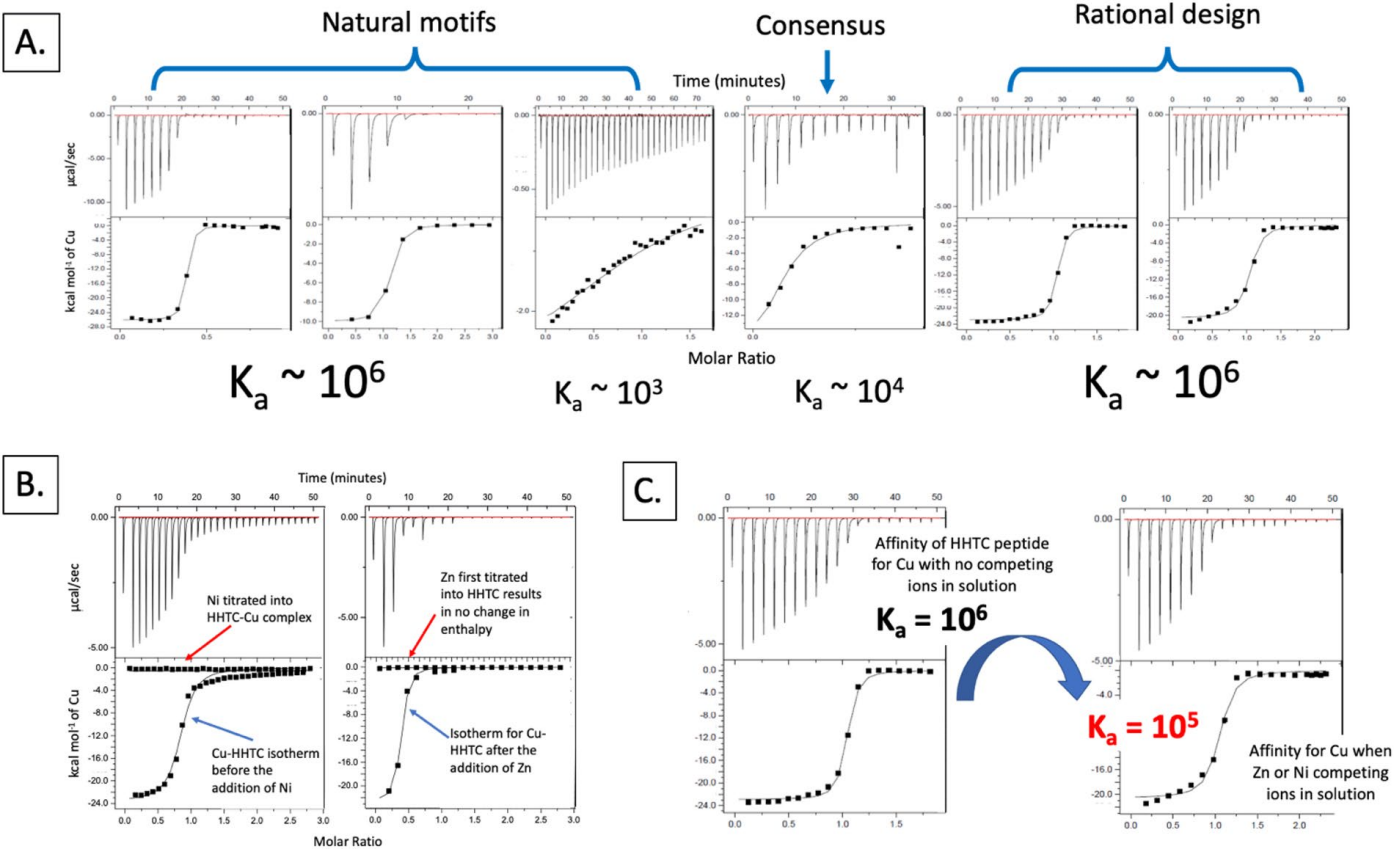
(**A**) Raw data and isotherms for tested peptides and Cu. The natural HypB peptides had a range of affinities with K_a_ = (2.37 ± 0.7) × 10^6^ M^−1^ and (1.30 ± 0.07) × 10^6^ M^−1^, for HypB1 and HypB2, respectively. The HypB peptide was tested with and without leading and trailing residues and showed that the addition or absence of the amino acids at the N- and C- termini did not lead to an appreciable difference in Cu binding affinities. The consensus sequence Cu-02 exhibited a mid-range affinity with K_a_ = (9.89 ± 2.18) × 10^5^ M^−1^ and the CZB-7 peptide had low affinity with K_a_ = (7.78 ± 1.25) × 10^3^ M^−1^. The designer peptides showed comparable affinities to the HypB motifs with HHTC K_a_ = (1.89 ± 0.3) × 10^6^ M^−1^ and CHSK K_a_ = (1.28 ± 0.3) × 10^6^ M^−1^. (**B**) In this experiment, Cu was first titrated into HHTC and an isotherm was calculated based on changes in enthalpy. Ni was then titrated into the HHTC-Cu complex and this resulted in no isotherm calculated for Ni indicating that Cu was not displaced from the peptide. Additionally, raw data and isotherm for CHSK solution containing Zn with added Cu where Zn was first titrated into CHSK and revealed no changes in enthalpy. Cu was then titrated into the sample cell containing CHSK + Zn and this resulted in an isotherm for Cu. (**C**) Raw data and isotherm showing binding affinity of HHTC for Cu in the absence and presence of the competing ion, Zn.

The natural peptides showed a range of affinities for Cu as shown in Fig. 1A and Table 1. The binding affinities for the rationally-designed motifs HHTC and CHSK had high affinities for Cu and were comparable to the natural HypB peptides. Binding parameters for HHTC with Cu have been previously published and were conducted at pH 7 with ACES buffer^27^. At this pH, thermodynamic modeling and experimental data show that Cu is mostly precipitated (>99%) into a solid mineral Cu(OH)_2(s)_ phase^10^. Thus, we suspected that the reported binding parameters would be confounded by a change in enthalpy due to Cu dissociation from a solid mineral phase, rather than from Cu binding to the HHTC peptide. We conducted our binding experiments at pH 5.5, where 99.991% of Cu is predicted to remain as Cu^2+^. The published binding affinity for HHTC is K_a_ = (2.4 ± 0.5) × 10^6^ M^−1^. Our observed parameters for HHTC match these within error despite differences in experimental conditions. Additionally, the use of buffers with different heats of ionization, such as ACES and MES (used in this study) buffers, with *ΔH*_*ion*_ = 31.4 kJ/mol and 15.5 kJ/mol, respectively, did not appear to affect the measured enthalpies for Cu binding. These results indicate the previously published binding parameters accurately describe binding between Cu and the peptide under these conditions and are not due to proton transfer or Cu-mineral phase changes.

### Specificity of metal binding

E-waste components have multiple metals, and these remain in solution after removal of the scaffold material. In order to determine if our constructs were specific for Cu, we added competing metal ions to the peptide-Cu complex. Ni and Zn were chosen as the competing ions and are often competitors for the same ligand due to their similar electron configuration, ionic radius, valence, and/or molecular geometry. Similarly, Cu was added as the competing ion to solutions with other metal-peptide mixtures. Intrinsic binding parameters were first determined for each peptide with individual metal solutions then apparent binding parameters were obtained when a competing metal was titrated into the already-formed metal-peptide complex. Previously published binding parameters on the select peptides did not determine whether they were specific to or showed preference for Cu when competing with other ions in solution. Figure 1B shows isotherms for competition experiments with HHTC and Cu, where Ni was titrated into the HHTC-Cu complex and resulted in no observed change in heat. Additionally, Cu was titrated into the sample cell containing HHTC and Zn and revealed Cu but no Zn binding. From this, we concluded that Cu occupied all available binding sites and was not dissociated from the peptide when competing ions were introduced into the solution. In all cases where Zn or Ni was titrated into an already-formed peptide-Cu complex, there was no change in enthalpy when the competing metals were added to the solution, indicating that Cu was not replaced by the competing ions.

We then tested whether the peptides would bind to Zn or Ni only, and no isotherm was calculated in all cases, indicating the peptides did not bind these metals (Table 1). We subsequently titrated Cu into the peptide + Zn, or peptide + Ni solutions to determine if the affinity for Cu was retained when competing ions were in solution. The HypB peptides retained their affinity for Cu at the same or higher level when Ni or Zn was in solution while Cu affinity for the consensus motif Cu-02 was an order of magnitude lower when Zn was in solution and Cu affinity was lost when Ni was in solution (Table 1). The CZB-7 peptide that had previously shown a weak affinity for Cu at K_a_ = (7.78 ± 1.25) × 10^3^ M^−1^, did not bind Cu at all when the competing ions were in solution even though there was no appreciable binding to Zn or Ni, as no isotherm was calculated in these cases. In the case of the synthetic peptides, the affinities of HHTC and CHSK for Cu were lowered by an order of magnitude when Zn or Ni was in solution (Table 1). Additionally, the peptide CHSK showed the affinity for Cu was lost when Ni was the competing ion. The Cu isotherm for HHTC is shown after it was titrated into a solution containing Zn and HHTC (Fig. 1C).

It is a recognized phenomenon that a determinant of peptide selectivity for a divalent metal ion follows the Irving-Williams series^28^, where the stability constants (*i.e*., strength of bonds due to electrostatic interactions between molecules) for metal complexes with any set of ligands is: Mg^2+^  < Mn^2+^  < Fe^2+^  < Co^2+^  < Ni^2+^  < Cu^2+^ > Zn^2+^. If this is the case, then any peptide tested would preferentially bind Cu, and it would displace all of the other metals in the series, regardless of other metal-ligand specificity principles. To test whether the rational design approach was effective at predicting specific binding to other metals, or whether *any* peptide would bind Cu with high affinity, we assessed peptides that were designed to bind Zn. The Zn-binding peptide KDTK had a higher affinity for Cu than it did for the cognate metal, Zn (Table 1) and KDKD did not show any binding to Zn, however did bind Cu with low affinity. Thus, with these peptides, Cu bound with higher affinity by at least an order of magnitude than for the cognate metal, Zn.

### Binding parameters of modified synthetic peptides

**Figure 2:**
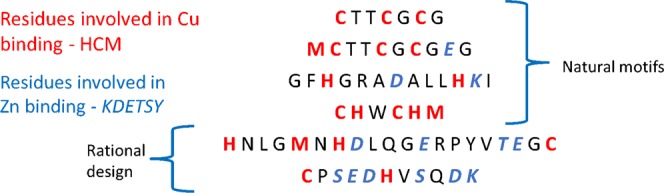
Primary amino acid sequences for natural peptides (HypB1, HypB2, CZB-8, Cu02) and those derived through rational design (HHTC, CHSK). Amino acids that are highly represented in Cu binding are shown in red, and those most implicated in Ni or Zn binding are shown in blue.

We observed that the rationally-derived peptides had a lower affinity for Cu when competing ions were in solution, despite there being no apparent binding to the competing ions, and this led us to further examine the designer peptides. We compared primary amino acid sequences for the natural and rationally-derived peptides and found that some of the latter contained residues that are highly represented in Ni and Zn binding sites (Fig. 2). Data mining studies show KDETSY (Lys-Asp-Glu-Thr-Ser-Tyr) amino acids are favored in Zn and Ni binding sites^29^ while 95% of residues in Cu binding sites are HCM (His-Cys-Met)^23^. Not surprisingly, we found that the natural motif from the HypB protein did not contain any of the Zn/Ni binding residues, while the peptides with low Cu-binding affinities such as CZB-7 and the rationally designed peptides did (Fig. 2).

To test whether the KDETSY residues were essential for retaining specificity for Cu by accommodating for molecular geometry, we assessed the binding parameters for HHTC and CHSK that had either the KDETSY residues omitted or replaced with non-interacting residues NLGQV (Table 2). The Cu binding affinities of the modified motifs were comparable to HypB and unmodified HHTC and CHSK affinities. An unpaired t-test between the original HHTC peptide and the one with KDETSY residues omitted (HHTC-Tr), revealed no significant difference (two-tailed P value = 0.2783) in intrinsic binding affinity for Cu. The HHTC-Tr peptide had an intrinsic binding affinity for Cu comparable to the unmodified peptide and retained its affinity for Cu when Zn or Ni were in solution. The HHTC peptide with the KDETSY residues replaced with NLGQV had a slightly higher affinity (but not quite statistically significant by conventional criteria, two-tailed P = 0.0621) for Cu than the original peptide with and this peptide retained its affinity for Cu when Zn or Ni was in solution. The binding affinity for CHSK, however was markedly different when non-binding amino acid residues were replaced or omitted. When the KDETSY residues were omitted from the peptide, CHSK-Tr Cu affinity was similar to the original CHSK, however affinity for Cu was lost when Ni or Zn were present in solution even though no binding isotherm was observed for Ni or Zn. When the CHSK peptide had the KDETSY residues replaced with the non-interacting NLGQV, binding affinity of CHSK-Re for Cu was lowered by an order of magnitude and this was only slightly less if Zn was in solution prior to the addition of Cu and completely lost if Ni was the competing ion.

**Table 2:** Intrinsic association constants (K_a_) for modified rational design peptides titrated with Cu, Zn, Ni, and apparent binding constants when titrated with Cu after Zn or Ni was in solution. Arrows Zn → Cu, Ni → Cu, indicate the association constants for Cu after Zn or Ni are in solution as competing ions. Peptides in tandem were not assessed for Ni or Zn affinity.

Type	Name	Amino acid sequence	Cu K_a_ [M^−1^]	Zn K_a_ [M^−1^]	Ni K_a_ [M^−1^]	Zn → Cu K_a_ [M^−1^]	Ni → Cu [M^−1^]
Rational design with KDETSY residues removed	HHTC Truncated	HNLGMNHLQGRPVTGC	(4.92 ± 2.17) × 10^6^	0	0	(1.67 ± 0.85) × 10^6^	(1.50 ± 0.69) × 10^6^
	CHSK Truncated	CPHVSQK	(1.08 ± 0.10) × 10^6^	0	0	0	0
Rational design with KDETSY residues replaced with NLGQV	HHTC Replaced	HNLGMNHVLQGNRPLVTQGC	(1.55 ± 0.21) × 10^6^	0	0	(3.99 ± 2.24) × 10^6^	(6.42 ± 3.76) × 106
	CHSK Replaced	CPNLGHVSQNK	(4.86 ± 0.83) × 10^5^	0	0	(3.34 ± 0.46) × 10^5^	0
2xHHTC Replaced in tandem	2x-HHTC-Re	HNLGMNHVHNLGMNHVLQGNRPLVTQGCLQGNRPLVTQGC	(3.73 ± 0.53) × 10^6^	—	—	—	—
3xHHTC Replaced in tandem	3x-HHTC-Re	HNLGMNHVLQGNRPLVTQGCHNLGMNHVLQGNRPLVTQGCHNLGMNHVLQGNRPLVTQGC	(1.50 ± 0.05) × 10^6^	—	—	—	—

### Tandem metal-binding motifs and linear increase in metal binding

**Figure 3:**
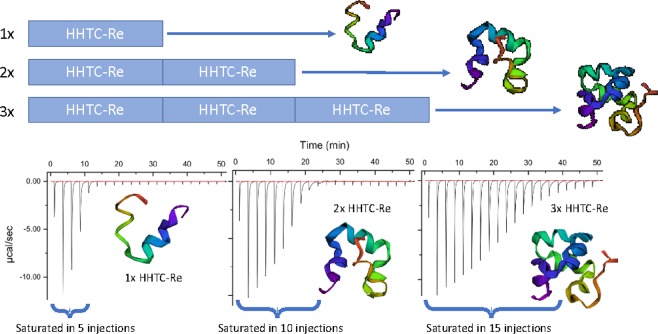
ITC experiment to determine binding parameters for peptides 1x-, 2x-, and 3x-HHTC-Re and visual representation of the peptide molecules predicted by the Zhang lab QUARK a*b initio* program. The binding affinity K_a_ reveals strong Cu binding for all tested peptides and a trend is observed that suggests the 2x and 3x peptides do bind 2 and 3 times more Cu, respectively.

Previous studies and our own observations determined that the peptides we tested all had a 1:1 stoichiometry to Cu. In an applied setting it would be ideal to bind more than one metal atom per peptide molecule, thus we tested whether having more than one metal binding motif in each peptide molecule would lead to a stoichiometric increase in Cu binding. Since we ultimately seek to move away from natural proteins with native metal co-factors, we chose to continue with the rationally designed peptide, HHTC, because it remained robust when non-binding amino acids were excluded or replaced with amino acids that did not interact with competing ions Ni or Zn. We modeled^30^ and constructed HHTC-Re motifs designed to have tandem repeats in series as single (1x-HHTC-Re), double (2x-HHTC-Re), and triple (3x-HHTC-Re) peptide molecules and assessed them for their binding parameters. We assessed these for Cu binding parameters with ITC and binding affinity K_a_ reveals strong Cu binding for the all of the peptides (Table 2). Additionally, a trend is observed that suggests the 2x and 3x peptides do bind 2 and 3 times more Cu, respectively as a linear increase is observed as we add more motifs in series and thus, we can bind multiple metal ions per peptide molecule (Fig. 3).

### Functionalizing mycelium surface with metal-binding peptides containing chitin-binding domain

When designing our biofilter, we had two guiding questions in mind: how can we best bind metals on a molecular level, and how can we create a platform for functionalizing mycelial material? We also considered the advantages of using mycelia in comparison to previous efforts, such as flagella-based or cellulose filtration tools^31,32^. One of the biggest benefits of using mycelium material on this NASA-funded project is that it leverages the concept of economies of scale, and presents a feasible option for scale-up of our technology to a level that could be successfully implemented on a space mission or on Earth in developing countries with poor access to clean water. Fungi are capable of growth on diverse biomass types, and grow at a rate that is unparalleled by other biological agents used in synthetic biology applications today^33^.

Using fungal mycelium as an immobile substrate, we designed a cost-effective, scalable, biodegradable filtration system for metal recovery from aqueous solutions using copper as a proof of concept. We assessed the feasibility of metal sequestration using a functionalized mycelia by treating the fungal surface with peptides containing metal-binding motifs in tandem repeats containing a chitin-binding domain that could bind to the solid mycelial surface of *Gandoderma lucidum. G. lucidum* has been described in literature for heavy metal binding, however this strain was not chosen for the innate metal-binding abilities, rather because it was shown to be a suitable candidate for the objectives of our overall project; these include ease of growth on diverse substrates, under different temperature regimes, and in environments that would be representative of those found in space-exploration applications. Please refer to the iGEM Stanford-Brown-RISD team website: for the characterization of this and other fungal strains: http://2018.igem.org/Team:Stanford-Brown-RISD/Experiments.

Mycelium, the vegetative structure of a fungus that is analogous to the root system of most plants, can branch out and bind various substrates to fill molds in different shapes, thus making it a good candidate for water filtration applications.

**Figure 4:**
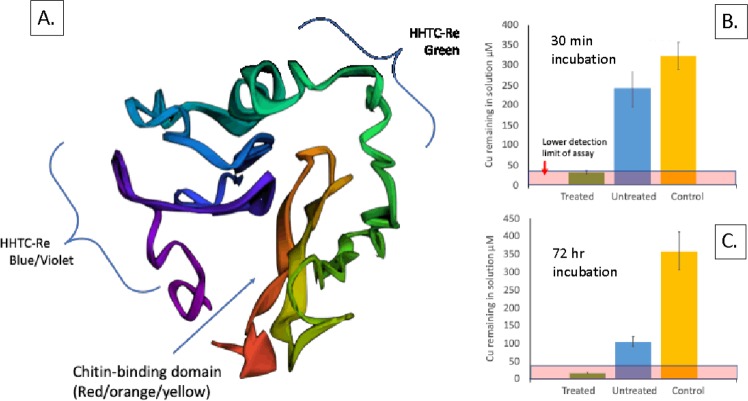
(**A**) QUARK *ab initio* model of 2x-HHTC-Re with chitin binding domain. Domains within the fusion protein have been annotated to display the conformation and spatial orientation. (**B**) Cu (µM) remaining in solution for n = 3 samples after incubation with mycelium treated or not treated with CBD-2xHHTC-Re for 30 minutes and (**C**) after 72 hours. The Cu concentration in the initial copper solution was 325 (+/−25) µM Cu. Control reactions contained Cu solution only. All experiments were conducted in triplicate. The amount of Cu adsorbed was calculated by taking the difference between the initial Cu in system and the remaining Cu after incubation with treated or untreated mycelium. Cu is below detection limits for the treated filters after 30 minutes and 72 hours. After 30 minutes of tangential flow, the untreated mycelium adsorbed about 23% of the copper in solution, while the treated mycelium (filter prototype) was able to sequester ~92% of the available Cu in solution.

**Figure 5:**
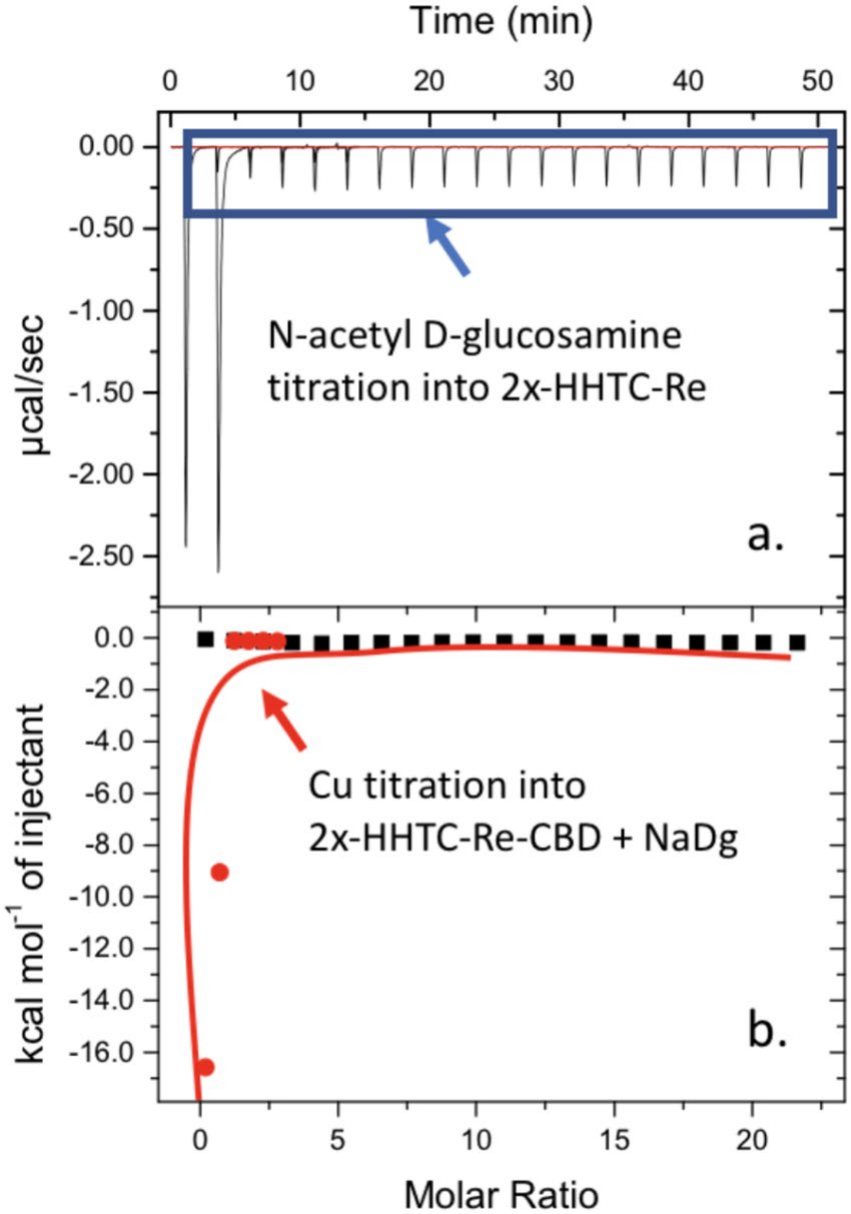
ITC raw data and isotherm for experiment to assess binding affinity of fusion protein CBD-2x-HHTC-Re for chitin (represented by *N*-acetyl *D*-glucosamine) and Cu. Motifs retained affinity for chitin monomers in the presence of Cu, and retained affinity for Cu in the presence of chitin (monomers).

The peptides were designed to contain a chitin-binding domain (CBD), flexible linkers GSGGSG, and 2x-HHTC-Re (Fig. 4A). After confirming the copper binding of the individual HHTC-Re x n peptides (see previous section), we then needed to assess whether our fusion protein could bind copper and chitin, and whether it could do so when already saturated with the other substrate. Figure 5 depicts two experiments a) Raw data and b) isotherm for 2x-HHTC-Re-CBD + NaDg and Cu. In this experiment, *N*-acetyl *D*-glucosamine (“NaDg”; analogous to a chitin monomer and widely used in the literature for assessing chitin binding) was first titrated into 2x-HHTC-Re-CBD and no isotherm was calculated because binding sites were not saturated by the ligand. Cu was then titrated into the 2x-HHTC-Re-CBD + NaDg complex and this resulted in a Cu affinity at K_a_ = (7.61 ± 1.49) × 10^6^ M^−1^ that is an order of magnitude higher than 2x-HHTC-Re (no CBD) with K_a_ = (3.73 ± 0.53) × 10^5^ M^−1^ and comparable to 2x-HHTC-Re-CBD without bound NaDg with K_a_ = (1.55 ± 0.21) × 10^6^ M^−1^. Data show 20 1 µL injections. 2x-HHTC-Re-CBD was selected as the candidate for testing because it displayed the most consistent and strong results during protein purification procedures and seemed most promising for downstream applications (such as our filter). Future work will focus on optimizing protein binding parameters to the chitin substrate and creating new filtration prototypes.

Mycelium treated with and without 0.45 mM 2x-HHTC-Re-CBD Cu/chitin-binding peptides were incubated with a solution containing Cu to determine the amount of Cu sequestered by the mycelium. The Cu concentration in our starting solution was 325 (+/−25) µM Cu and after an incubation with the untreated mycelium we observed that the remaining solution was 250 (+/−50) µM Cu, thus the untreated mycelium adsorbed approximately 23% of the Cu available in solution (Fig. 4B,C). In contrast, after a 30-minute incubation, the mycelium treated with the CBD-2x-HHTC-Re motifs removed Cu to below detection limits thus sequestering at least 92% of the available Cu in solution. Incubation for 72 hours revealed that the untreated mycelium removed about 70% of Cu in solution while the treated mycelium removed all Cu from solution within the detection limits of the assay. Future work in this area will focus on characterization and optimization of the fungal mycelial surfaces and metal-binding peptides.

## Discussion

In this work, we compared binding affinity and specificity of naturally-occurring metal-binding motifs and peptides derived *in silico* and found that they had comparable binding parameters. Cu is an essential element in biology with roles as a cofactor in a wide range of proteins and differences in binding affinities are due to the function of the metalloprotein they are derived from. Copper is a highly reactive element that can cause oxidative damage if not tightly controlled and regulated by the cellular machinery thus, Cu proteins are characterized by high affinity for their cognate metal. When applying the rational design approach for peptide engineering, an important consideration is that the algorithms used to predict peptide binding partners rely on protein databases to characterize the molecular geometry of a cognate metal in a metalloprotein. The rationally-designed motifs showed comparable affinities to the natural peptides because they themselves are derived from known Cu structures in Protein Data Bank^29^.

The algorithms used by previous researchers build from known metalloprotein structures to take short motifs and put them together into one peptide that fulfills the molecular geometry of the target metal^27^. A key feature of these previous approaches is that only metal-peptide complexes for which there is crystal structure data are used, thus limiting the computational approach to metals with known biological functions and excluding metals that have not been characterized for biological interactions. It is possible to model interacting ligands with amino acid binding partners represented by individual residue side chains and without the need for the short motifs obtained through PDB that add rigidity to the peptides predicted to bind Cu. For example, it was found that while softer ligands such as cysteine and methionine have to be modeled using bulkier representations (*e.g*., the whole side chain of the residue), harder ligands can be represented by simply using carboxyl groups, amines, and alcohols in computational models^34^. This approach to peptide prediction achieves a similar level of accuracy to using the whole amino acid molecule, while greatly reducing the computational requirements.

The rationally-designed HHTC and CHSK peptides give insight into the types of binding a metal can participate in. Both fulfill the preferred geometry of Cu, square planar, but with very different binding residues that suggest a certain fluidity in binding principles with regard to the ligands involved. Both tested peptides have non-canonical binding partners (threonine in HHTC, and serine and lysine in CHSK) as the residues that coordinate a Cu atom yet they each bind selectively and with similar affinity despite having vastly different primary sequences. Their affinity for Cu was enhanced when removing or replacing the amino acid residues (KDETSY) that were presumed to interact with competing metal ions. It is possible that this observed phenomenon was due to a more favorable fit around the Cu ion. An interesting phenomenon was observed when the Cu-02 and modified CHSK motifs, which lack the Zn and Ni binding residues, both lost affinity for Cu when Ni was in solution. While the peptide was designed for the square planar molecular geometry of Cu, Ni can bind in octahedral molecular geometry which can also accommodate square planar molecular geometry, thus making these peptides susceptible to competitive inhibition^35^.

In this sense, the algorithm used to create these peptides and predict optimal binding for Cu, is limited by having to use only PDB structures. For future work in rational design, it is essential that all of the residues in a peptide, not only the ligands that interact with the metal ion, be considered for specificity since interaction with competing ions will reduce affinity for the cognate metal.

The rational design peptides KDKD and KDTK, that were designed to bind Zn did bind Cu with more affinity than for their cognate metal and thus conform to the Irving-Williams stability series principles. While no S-containing residues are present in the peptide sequences (cysteine, methionine), both sequences tested contain the hydroxyl-containing threonine resides that can interact with Cu. It would be curious to test whether removal of these residues would affect their affinity to Zn while limiting the electrostatic interactions with Cu. Additionally, since Cu is predicted to form the most stable complexes, future approaches in metal sequestration from mixed solutions should follow the order of the stability of complexes as stated in the Irving-Williams series.

To develop a pathway to implemented metal recovery, we explored the feasibility of metal attenuation with our engineered peptides and functionalized fungal mycelia and found that incubation with metal-binding peptides containing a chitin binding domain increased the amount of Cu removed from solution. Previous studies have characterized the adsorptive properties of mycelium and they found that differences in metal-binding capacity for Cu was directly related to the cation exchange capacity at the mycelial surface of individual fungal species^36^. Thus, there is an inherent ability for mycelium to attenuate heavy metals despite the hydrophobicity of the chitin substrate. This property is further enhanced by treatment with our peptide constructs that turn an otherwise hydrophobic surface into a functionalized adsorptive surface that can interact with dissolved ions in aqueous solutions. While cellulose provides an alternative substrate and cellulose-binding motifs are well-known, the production of nanocellulose requires substantial inputs of glucose, and other forms of cellulose incur agricultural costs^37^. Thus, we believe fungal mycelia provide a better alternative in most situations including bioremediation.

Future work in metal-binding peptide design should introduce molecular geometries for metals that have no role in biology but are of geopolitical importance such as the lanthanide series and platinum group elements. These metals do not have biological roles, however, by calculating association energies between the metals and amino acid residue side chains, the free energies can be approximated, and peptide partners predicted. If no known molecular binding partners in available amino acid side chains exist, then using synthetic amino acids with residues that have the ability to bind metal ligands can be another approach at targeting these metals.

In conclusion, we found that motifs developed through rational design by applying quantum mechanical methods that account for complexation energies of the elemental binding partners and molecular geometry of the cognate metal, not only show high affinity for the cognate metal, but they show specificity and discrimination against other metal ions that would-be competitors for the same binding sites. Previous computational models used to predict metal-peptide complexes use known crystal structure data, however this limits peptide models to metals with biological function and known ligands. It is possible to model interacting ligands *de novo* with binding partners represented by using individual residue side chains as discreet binding partners and this approach can be used to design peptides to recover other metals such as rare earth or platinum group elements for biomining/recycling.

## Methods

### Peptide synthesis

Peptides were synthesized by Elim Biopharmaceuticals (Hayward, CA, USA), purified by HPLC with (H)Cl as the counter ion, and provided as a lyophilized powder. Purity was > 98% and verified through mass-spectrometry. Peptides were modified to have N-terminal acetylation and C-terminal amidation to avoid having a charged peptide. Peptides were reconstituted in 10 mM 2-(N-morpholino)-ethanesulfonic acid (MES) buffer pH 5.5 and concentrations determined through spectrophotometry with the Pierce™ BCA Assay.

### Fusion protein production and purification

The DNA sequences encoding the 2x-HHTC-Re protein constructs containing an intein self-cleavage site, hexahistidine and Lumio ® detection tags, were commercially synthesized (IDT, gBlocks®) and were assembled into the PSB1C3 iGEM backbone using Gibson Assembly (NEB), transformed NEB DH5a® competent *E. coli* for plasmid construction and transformations plated on chloramphenicol selective LB plates. The transformations were incubated at 37 °C overnight and DNA constructs confirmed in select colonies using verification primers and colony PCR. Plasmids from sequence-verified clones were then transformed into the T7 Express® *E. coli* (NEB) protein production strain. His-tag purification on crude cell extract from our colonies using ThermoFisher HisPur® Ni-NTA spin columns. The standard protein purification protocol was modified by introducing a buffer containing 50 mM DTT for on-site cleavage, so the desired fusion protein could be eluted. We then performed a BCA Assay to determine our total protein concentration in each elution. Following protein de-salting with Amcon® filter spin columns, we confirmed the presence of target protein in the final elution using ThermoFisher Scientific Lumio® Tag Detection Kit.

### Mycelium biomass production

To test the peptides, the monokaryon form of *Ganoderma lucidum* from USDA Forest Products Laboratory culture collection was grown in plates containing liquid Potato Dextrose Yeast Agar (PDYA), composed of 2.0% dextrose, 0.10% potato extract, 0.15% yeast extract, and 97.50% water at 30 °C for 14 days. Pure mycelium material was isolated, cut into strips, pressed into thin uniform sheets, dried at 120 °C for three hours to kill the fungus, and then cut into uniform 1 cm^2^ pieces to be used as filter prototypes. We tested growth rates on varied substrates to determine which could be used with minimal added growth medium. Noteworthy substrates of environmental relevance were sawdust, lawn clippings, and used coffee grounds and other forms of food waste. The standardized substrate on which we tested different conditions was potato dextrose yeast agar (PDYA)s which provides optimal nutrients for the fungus without providing excess nutrients that would encourage bacterial growth.

#### Phen green SK assay

For Cu adsorption estimation, Phen Green SK dye (Invitrogen, USA) was prepared in a stock solution of 28 µM in 0.9% PBS. PGSK fluorescence is quenched in the presence of Cu and is proportional to the amount of Cu in a solution. 200 µL of this stock solution was added to each well in a 96-well plate to which 25 µL of sample was added. All experiments were done in triplicate and the reported concentrations are the average of n = 3 experimental replicates. A standard curve was generated for Cu and PGSK and gave approximations of the amounts of Cu in the tested solutions.

### Bulk adsorption experiments

All experiments were conducted in triplicate. One cm^2^ pieces of mycelium were either treated with purified protein 0.45 mM CBD-2xHHTC in 10 mM MES buffer pH 5.5 or with buffer only in shake flasks for 24 hours. Treated and untreated mycelium were placed in 3 mL of 0.25 mM Cu solution and samples taken at 30 minutes and at 72 hours. The incubation medium was analyzed for the remaining Cu in solution. The amount of Cu adsorbed was calculated by taking the difference between the initial Cu in system and the remaining Cu after incubation with treated or untreated mycelium.

### Isothermal titration calorimetry

Isothermal titration calorimetry (ITC) was used to determine the association equilibrium constant (K_a_). The instrument used to obtain measurements was a Malvern MicroCal iTC200 in the Space Biosciences Division at NASA Ames Research Center, Mountain View, CA. Instrument performance was verified by running the standard Ca-EDTA titration kit available from Malvern Analytical. All binding parameters for the test were within the specifications determined by the manufacturer.

The buffer chosen for the ITC experiments was 10 mM, MES buffer. It was chosen because it has been shown to not cause metal ion interference as a result of complexation or amine oxidation, is stable through the entire range of pH 3–11, and has a stable pK_a_ over a relatively wide temperature range (15 °C–45 °C)^38,39^. Buffer pH for peptides and metal solutions was 5.5 to prevent metal precipitation. Metal chloride salts were used as the source of metal ions, and speciation was verified through thermodynamic modeling using Visual Minteq. 3.0^40^.

Concentrations for metal stock solutions were determined with the Thermo iCAP 7400 Inductively Coupled Plasma Optical Emission Spectrometer (ICP-OES) at the University of California Santa Cruz, Marine Analytical Laboratory.

To measure Cu binding to our peptide, the metal solution was prepared from cupric chloride, dihydrate, crystal, BAKER ANALYZED™ A.C.S. Reagent, J.T. Baker™. The peptides were used without further purification. The peptide solutions were prepared by dissolving a weighed amount of the lyophilized powder in 10 mM MES prepared from Alfa Aesar™ MES, 0.2 M buffer soln., pH 5.5. pH. Metal solutions were prepared by dissolving a weighed amount of the pure metal chloride salts into the same stock MES buffer that was used to prepare the peptide solution to minimize the effect of the heat of dilution/mixing when measuring the samples.

The ITC experiments were run at 25 °C and set to deliver 20, 0.5–1 µL injections at 150 sec intervals. Titrate and titrant solutions were de-gassed prior to loading into calorimeter cell and injection syringe. The procedure involved titrating (Cu, Zn, Ni)-Cl_2_ in excess by 10–20 times the concentration of the cognate motif. Typically, the peptide solutions were prepared to 0.5 mM, and the metal salts were at 2.4–6.4 mM concentrations. In some cases of low affinity, or when no saturation of the metal-binding peptide was observed, up to 100 times the concentration of metal was used. Metal-chloride salts were chosen because they remain as dissolved ions with chloride as the counter ion. This was complementary to the peptide conditions where chloride (HCl) was used as the counter ion during purification. The experiments were run such that the metal solution in the syringe was titrated into the peptide solution in the cell. Raw data were corrected by subtracting the heats of dilution. Integrated heat data were fit with a one-site binding model using the Origin-7™ software provided with the MicroCal iTC200. The “best-fit” parameters resulting from the nonlinear regression fit of these data are also shown in the figures.

### Controls and heat of injections, heat of dilution

The mixing and dilution effects for the ITC experiments were minimized by using the same buffer for the peptide and metal salt preparations. Heat of dilution was determined by three titration experiments where 1) metal chloride (ligand) solution was titrated into buffer in the sample cell, 2) buffer was injected from the syringe into the peptide-buffer solution in the sample cell, and 3) buffer was titrated into buffer only in the sample cell. Metal-chloride titrations into the sample cell with blank buffer released heats comparable to blank buffer mixing where buffer-buffer titrations released 0.02 μcal/sec per injection, 5 mM CuCl_2_ = 0.08 μcal/sec, 2.6 mM NiCl_2_ = 0.05 μcal/sec. Heat of dilution/mixing for 6.4 mM ZnCl_2_ was measured at 1.0 μcal/sec per injection and up to 15 μcal/sec for 64 mM ZnCl_2_. In cases where heat of dilution/mixing caused a high background, the blank values were subtracted from the raw data prior to model isotherm fits.

The heat of ionization of the buffer due to the release or uptake of protons during binding from the buffer conjugate base was determined to be negligible thus we did not correct for heat of ionization of the MES buffer^41^.
